# Innovations in intracranial aneurysm treatment: a pilot study on the Choydar flow diverter

**DOI:** 10.3389/fneur.2024.1413681

**Published:** 2024-07-04

**Authors:** Changya Liu, Yike Cai, Chunguang Zang, Kaikai Guo, Xuebin Hu

**Affiliations:** ^1^Department of Neurosurgery, Union Hospital, Tongji Medical College, Huazhong University of Science and Technology, Wuhan, Hubei, China; ^2^Department of Emergency, Longhua Hospital, Shanghai University of Traditional Chinese Medicine, Shanghai, China; ^3^Department of Neurosurgery, Wuhan Red Cross Hospital, Wuhan, Hubei, China

**Keywords:** Choydar flow diverter, FD device, endovascular, intracranial aneurysms, efficacy and safety

## Abstract

**Background:**

The flow diverter (FD) has emerged as a promising treatment option for intracranial aneurysms. Recently, a novel flow-diverting stent, the Choydar FD device, has been developed within our nation.

**Objective:**

To introduce the newly developed Choydar FD device and present our preliminary clinical experience with its application in the treatment of intracranial aneurysms.

**Methods:**

A total of 23 patients with 23 unruptured intracranial aneurysms, comprising 20 (87.0%) aneurysms located at the internal carotid artery and 3 (13.0%) at the vertebral artery, were treated with the Choydar FD device between December 2021 and April 2022. Patient baseline data, clinical and angiographic outcomes were collected and analyzed.

**Results:**

The Choydar FD device was successfully deployed in all patients (100%), with 18 aneurysms (78.3%) additionally treated with coils. One patient experienced an ischemic event with sensory disturbance during the perioperative period. At the 1-year follow-up, all patients demonstrated good clinical outcomes. Of the 23 aneurysms with available angiographic follow-up, 22 (95.7%) achieved complete occlusion, and one patient exhibited in-stent stenosis without neurological deficits.

**Conclusion:**

The initial clinical results of the Choydar FD device are encouraging, and it appears to be a useful option for treating intracranial aneurysms with acceptable efficacy and safety. Future studies with larger sample sizes and longer follow-up durations are warranted to validate these findings.

## Introduction

In recent years, the deployment of flow diverter (FD) devices has been recognized as a reliable endovascular treatment modality for intracranial aneurysms (1). Acquiring a comprehensive understanding of the technicalities and functionalities of FD device could aid in the identification of suitable lesions and the enhancement of procedural efficacy. The fundamental principle underlying the application of FD device entails redirecting the blood flow away from the aneurysm in a direction aligned with the longitudinal axis of the parent artery (2). The technique not only reduces the velocity of blood flow within the aneurysm and increases blood viscosity, leading to thrombosis within the aneurysm and successful treatment of certain lesions (3), but also facilitates endothelial cell proliferation while maintaining vessel patency (4), promoting the vascular reconstruction of the parent artery. Currently, several FD devices have been developed, implemented in clinical contexts, and proven to be both safe and efficacious (5, 6). Nevertheless, given the continuous innovations in materials application and structural designs of FD devices, alongside the sustained deepening of clinical use, further investigative efforts are necessary to propel the advancement and optimization of FD device applications. This study aims to provide an initial assessment of the Choydar Flow Diverter (CED Medtech, Jiangsu, China), a novel FD device, and to evaluate its effectiveness and safety in clinical applications.

## Materials and methods

This research is part of a prospective, multicenter, single-arm clinical trial conducted in our country, aimed at assessing the efficacy and safety of a blood flow diversion device for patients with intracranial aneurysms. The ethical review board of our institution granted approval for this study (Approval No. 2021-0947). Additionally, it was officially registered on the China Clinical Trial Registry platform (www.chictr.org.cn, Registration No. ChiCTR2100051153). The protocol of the study followed Good Clinical Practice and the Declaration of Helsinki.

### Patients

Between December 2021 and April 2022, patients diagnosed with intracranial aneurysms at our institution, who consented to undergo treatment with the Choydar FD device, were recruited for this study. The diagnosis was established, and the aneurysms’ morphology and location were accurately identified using Computer Tomography Angiography (CTA) or Digital Subtraction Angiography (DSA). The decision to proceed with either surgical or endovascular treatment was made following a comprehensive multidisciplinary consultation, ensuring a personalized treatment strategy tailored to each patient’s specific clinical scenario. Written informed consent was collected from all patients before the procedure.

### Inclusion and exclusion criteria

The application of the Choydar FD device was subject to specific inclusion and exclusion criteria.

Inclusion criteria were based on the following factors:

1. Participants must be aged 18 years or older.
2. Unruptured and untreated wide neck intracranial aneurysms confirmed by imaging as CTA or DSA, the locations of aneurysms include internal carotid artery, middle cerebral artery, anterior cerebral artery, posterior cerebral artery, and vertebral artery. A wide-necked aneurysm was defined as having a dome-to-neck ratio < 2 or a neck width of at least 4 mm.
3. Treatment was planned using the Choydar FD alone or in conjunction with coiling.
4. The diameter of the parent arteries ranged between 1.5 and 6.5 mm.
5. Baseline modified Rankin Scale (mRS) ≤ 2.
6. Written informed consent signed by the patients and their legal guardian.

The exclusion criteria include:

1. Requirement for embolization materials other than the Choydar FD and coils.
2. Stroke or ruptured aneurysm in the past 3 months.
3. Known platelet dysfunction or a platelet count < 100,000 cells/mm^3^.
4. Known adverse reaction to prasugrel or heparin.
5. Known allergy or intolerance to aspirin, clopidogrel, contrast, or anesthesia.
6. Kidney disease (creatinine > 2.5 mg/dL); baseline mRS ≥ 3; life expectancy < 12 months.
7. Patient refusal to use the FD device.
8. Pregnant or breastfeeding individuals.

### Characteristics of the Choydar FD device

**Figure 1:**
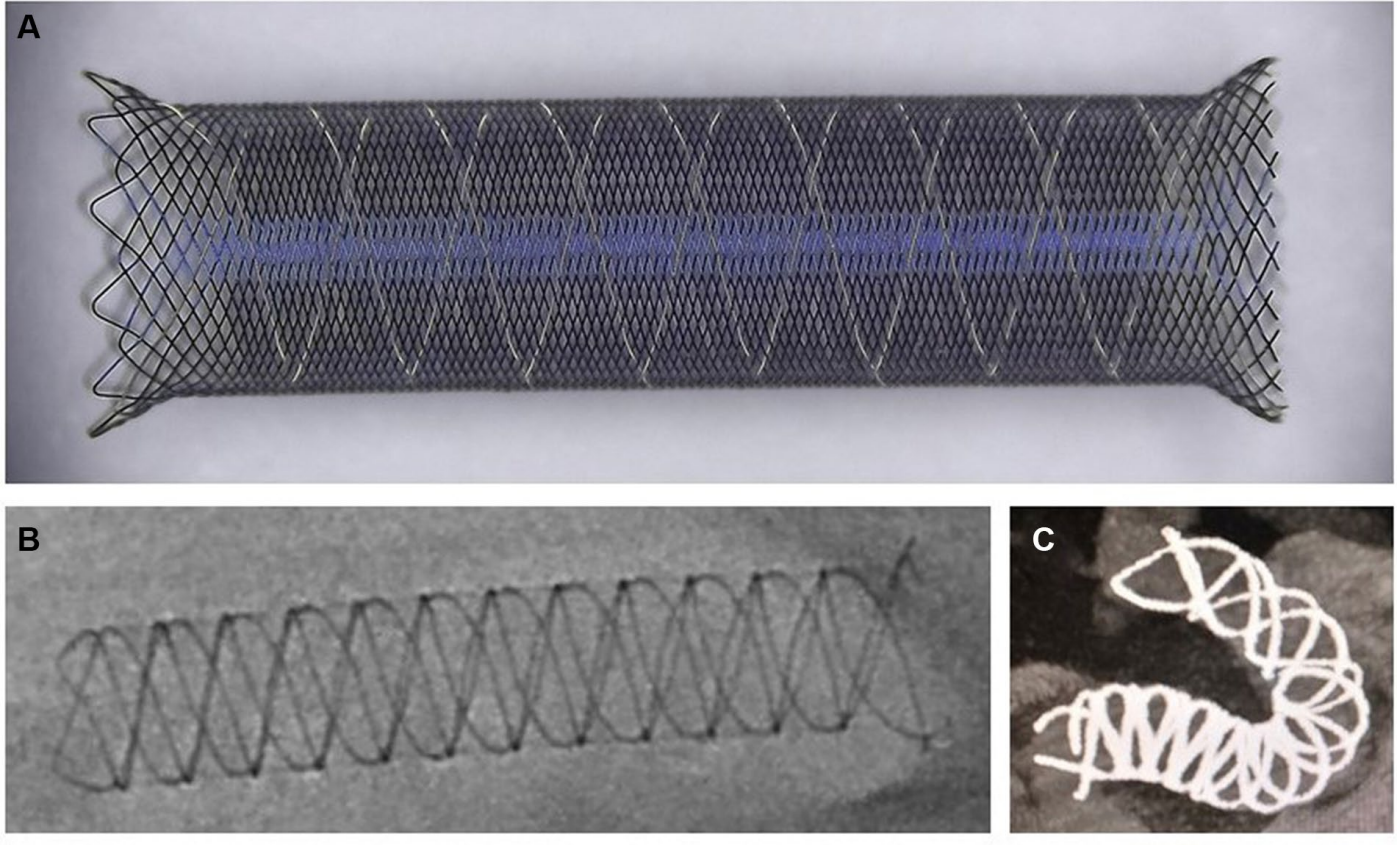
Schematic representation of the Choydar FD device. **(A)** The *ex vivo* released state of the Choydar FD device. A closed-cell structure situated at the left side (distal edge) of the stent; **(B)** Fully open state of the Choydar FD device through radiographic imaging; **(C)** Fully deployed state of the Choydar FD device through Dyna CT imaging.

The Choydar FD device, with its braided tubular stent configuration, is produced in two models: the first, with 48 nitinol wires, suitable for vascular diameters of 1.5 to 5.0 mm, and the second, with 72 nitinol wires, designed for 5.0 to 6.5 mm diameters. It is offered in nominal diameters from 2.0 to 6.5 mm and lengths between 10 and 55 mm. Each variant features a radiopaque structure made of four platinum helical strands intertwined with nitinol, starting from the device’s distal side and extending along the axis to improve X-ray visibility throughout its profile. This “edge-to-edge” strand view facilitates verification of the device’s alignment with the vessel wall. The Choydar’s metal coverage ranges from 30% to 35%, depending on the device’s size relative to the parent artery’s diameter. Uniquely, the device’s distal end adopts a closed-cell structure, distinguishing it from prior FD models. Figure 1 schematically illustrates the Choydar FD device, and a comparison of flow diverters’ structural characteristics is presented in Table 1.

**Table 1:** Structural characteristics comparison of flow diverters.

	Pipeline Classic and Pipeline Flex	Pipeline Shield	Surpass Streamline and Surpass Evolve	FRED	FRED Jr.	Silk and Silk +	Silk Vista Baby	p64	p48	Tubridge	Choydar
Material	Platinum, tungsen, cobalt, chromium	Platinum, tungsten, cobalt, chromium, nitinol	Cobalt, chromium, platinum, tungsten	Nitinol, titanium	Nitinol, titanium	Nitinol, titanium, platinum	Nitinol, titanium, platinum	Nitinol, titanium	Nitinol, titanium	Nitinol, titanium, platinum, iridium	Nitinol, titanium, platinum, tungsten
Braids number	48	48	48/72/96	64	52	48	48	64	48	64	48/72
Delivery catheter ID (inch)	0.027	0.027	0.027	0.027	0.021	0.021	0.017	0.027	0.021	0.029	0.017
											0.021
						0.023					0.027
			0.040			0.025					0.033
Resheath ability	Yes (Pipeline Flex)	Yes	Yes	Yes	Yes	Yes	Yes	Yes	Yes	Yes	Yes

FRED, Flow Redirection Endoluminal Device; ID, Internal diameter.

### Endovascular procedure

Prior to the intervention, pre-procedural imaging was employed to ascertain critical parameters such as the optimal working projection, the target vessel’s diameter, and the appropriate diameter and length of the FD device. A multidisciplinary team evaluated the patients to decide on the most suitable treatment strategy, considering factors like the choice of FD stent type and the potential incorporation of coiling within the aneurysm sac. Given the unconstrained device’s diameter is approximately 0.25 mm greater than its nominal diameter, selecting a slightly undersized stent was considered acceptable. The procedure was executed using either a single-plane or biplane angiographic system (Artis Zee, SIEMENS, Germany) under general anesthesia.

The Choydar FD device features a sophisticated delivery system designed, and the selection of the microcatheter for the device delivery is contingent upon the device’s diameter. Specifically:

For devices with diameters ranging from 2.0 to 3.0 mm, a microcatheter with a 0.017-in internal diameter (ID) is required. Devices with diameters between 3.25 and 3.75 mm necessitate a microcatheter with a 0.021-in ID. Devices with diameters from 4.0 mm to 5.0 mm require a microcatheter with a 0.027-in ID. For devices with diameters ranging from 5.25 to 6.5 mm, a 0.033-in ID microcatheter is needed. Details of the Choydar FD’s specifications are delineated in Table 2.

**Table 2:** Model specification sheet of Choydar flow diverter.

Product model	*Ex vivo* released state diameter (mm)	In nominal vessel released state diameter (mm)	Available lengths (mm)	Compatible microcatheter ID (inch)
FD200-X/FD200-X-MC	2.5	2.0	10/13/15/20	0.017
FD250-X/FD250-X-MC	3.0	2.5	10/13/15/20/25/35	
FD300-X/FD300-X-MC	3.25	3.0	10/13/15/20/25/30/35/40/45	
FD325-X/FD325-X-MC	3.5	3.25	10/13/15/20/25/30/35/40/45	0.021
FD350-X/FD350-X-MC	3.75	3.5	13/15/20/25/30/35/40/45	
FD375-X/FD375-X-MC	4.0	3.75	13/15/20/25/30/35/40/45	
FD400-X/FD400-X-MC	4.25	4.0	15/20/25/30/35/45/55	0.027
FD425-X/FD425-X-MC	4.5	4.25	15/20/25/30/35/45/55	
FD450-X/FD450-X-MC	4.75	4.5	15/20/25/30/35/45/55	
FD475-X/FD475-X-MC	5.0	4.75	15/20/25/30/35/45/55	
FD500-X/FD500-X-MC	5.25	5.0	15/20/25/30/35/45/55	
FD525-X/FD525-X-MC	5.5	5.25	15/20/25/30/35/45/55	0.033
FD550-X/FD550-X-MC	5.75	5.5	20/25/30/35/45/55	
FD600-X/FD600-X-MC	6.5	6.0	30/35/45/55	
FD650-X/FD650-X-MC	7.0	6.5	30/35/45/55	

mm, millimeter; ID, Internal diameter.

The stent is delivered using a three-coaxial support system that includes a long sheath or guiding catheter, an intermediate catheter, and the stent microcatheter. The delivery process involves advancing the stent to a straight segment of the vessel past the distal landing zone of the parent artery. The microcatheter is then retracted to deploy the stent from its distal end, ensuring precise placement and optimal apposition to the vessel wall. Radiopaque markers along the stent enhance visibility under fluoroscopy, facilitating accurate deployment.

To implant the Choydar FD device, either a 6 Fr long sheath or an 8 Fr guiding catheter is inserted into the femoral artery. A distal access catheter is then coaxially guided to the internal carotid artery (ICA) for anterior circulation aneurysms or the vertebral artery (VA) for posterior circulation aneurysms, creating a stable conduit. Following this, a 60 IU/kg heparin bolus is administered. A microcatheter is navigated to the parent artery for FD deployment, and if coiling is necessary, another shaped microcatheter is directed to a suitable site within the aneurysm. The FD device is subsequently delivered to the targeted position through the microcatheter.

### Stent selection and deployment

The stent’s diameter is selected based on the diameter of the proximal vessel (near the aneurysm neck), ensuring it matches closely for an optimal fit. The length of the stent is determined to cover the aneurysm neck with 8–10 mm margins on either side. For larger or more complex aneurysms, a longer stent may be chosen to ensure full coverage and stability.

During the intervention, optimal practice entails advancing the stent to a comparatively straight segment of the vessel past the parent artery’s distal landing zone, then retracting the microcatheter to initiate deployment of the FD device from its distal end. The stent opens gradually, forming a “V” shape initially, and the FD and microcatheter are then collectively retracted to the distal landing zone for complete stent deployment. This process involves pushing the delivery wire forward while concurrently retracting the microcatheter at a controlled pace. Upon covering the aneurysm neck with the FD, the surgeon must assess its adherence to the vessel wall to execute any necessary adjustments. After full deployment, stent apposition is meticulously verified using multiple-position angiography, dual-volume imaging, or Dyna CT. In cases of malapposition, corrective measures such as microcatheter manipulation (7) or appropriate balloon dilatation can be employed. Throughout the procedure, the system’s tension is managed to avoid excessive force, ensuring a smooth and precise deployment.

### Antiplatelet therapy protocol

In this study, participants received pre-procedural antiplatelet therapy, consisting of 75 mg clopidogrel and 100 mg aspirin daily, initiated at least 5 days prior to the procedure. The assessment of each patient’s response to antiplatelet therapy was conducted, leading to preoperative adjustments in the dosages of aspirin and clopidogrel based on platelet function test outcomes. For those demonstrating resistance to clopidogrel, prasugrel (10 mg daily or a 30 mg loading dose) or ticagrelor (90 mg twice daily or a 180 mg loading dose) was administered. At the onset of stent deployment, an intravenous infusion of tirofiban (100 mL/5 mg, Grandpharma, China) was initiated at a dosage of 0.10 μg/(kg/min) and sustained for 24 h.

### Perioperative complications

The recording of perioperative complications, specifically focusing on hemorrhage and thromboembolic events, was undertaken in this study. Intraoperative thromboembolism was defined by evidence of stent thrombosis, reduced flow in the parent artery during angiography, or the non-visualization of distal arteries. Postoperative thromboembolic events were characterized by the emergence of new positive symptoms or signs that were validated by magnetic resonance imaging (MRI) or computed tomography (CT) scan. Intraoperative hemorrhage was detected by the extravasation of contrast medium during angiographic procedures. Postoperative hemorrhage was determined based on clinical deterioration and CT evidence of bleeding.

For angiographically detected thrombus or vessel occlusion, a microcatheter was navigated to the vicinity of the thrombus for the intra-arterial administration of tirofiban, with a maximum dose of 0.5 mg. Mechanical thrombectomy was considered based on specific patient conditions. Hemorrhage control strategies included the use of protamine for heparin reversal, the temporary cessation of antiplatelet medications, and, if necessary, coiling embolization or angioplasty to arrest the bleeding.

### Measurement of angiographic results and clinical outcomes

Immediate angiographic results were measured with the O’Kelly-Marotta (OKM) scale (8), with assessments performed by two experienced neuro-interventionists CL and XH. The OKM scale classifies angiographic occlusion into four grades: A (total filling), B (subtotal filling), C (entry remnant), and D (no filling).

Perioperative patient conditions were assessed using the National Institutes of Health Stroke Scale (NIHSS) and the mRS. Post-procedure, patients were observed in the neurosurgery intensive care unit (NICU) for 24 h, with neurological deterioration identified by an increase of at least 2 points in the NIHSS score.

In case of postoperative neurological symptoms, immediate CT and CTA scans were performed. For non-hemorrhagic conditions, intravenous tirofiban (0.15 μg/kg/min) was administered. Mechanical thrombectomy was utilized for parent artery occlusion, while hemorrhagic events led to the cessation of antiplatelet therapy and consideration of external ventricular drainage or hematoma evacuation by the neurosurgeon.

CT scan was routinely performed within 48 h after the operation. Clinical outcomes, measured by mRS scores at discharge and during follow-ups, categorized scores of 0–2 as favorable and 3–6 as adverse. Outpatient follow-ups were scheduled at 1, 3, 6, and 12 months, using mRS scores for evaluations. Angiographic follow-ups were performed with CTA at 6 months and DSA at 12 months, with annual DSA examinations recommended thereafter.

### Study outcomes

The primary outcome of this study was delineated as the assessment of angiographic imaging immediately post-procedure, coupled with the identification of any novel neurological symptoms arising during the perioperative period. The secondary outcome concentrated on evaluating both clinical and angiographic findings documented throughout the follow-up period.

### Data collection and statistical analysis

All the clinical and imaging data of the patients, including DSA, CT, and CTA imaging, were collected by two collaborators. SPSS version 26.0 (IBM SPSS Software, United States) was used to perform the statistical analysis of the data. Continuous variables were presented as medians (interquartile ranges), categorical variables were presented as counts and percentages (%). *p*-values < 0.05 were considered significant.

## Results

During the study period, a cohort consisting of 23 patients, each with a single aneurysm, was examined and treated. A total of 23 Choydar FD devices were employed in these cases. The majority of patients (82.6%) were female, with a mean age of 56.5 ± 11.1 years. Aneurysm locations included 12 in the ophthalmic segment of the internal carotid artery, 4 in the posterior communicating segment, 4 in the carotid-cavernous segment, and 3 in the vertebral artery (V4 segment). The mean aneurysm length diameter was 8.4 ± 6.3 mm, and the mean neck width was 5.3 ± 2.1 mm. The baseline characteristics are summarized in Table 3.

**Table 3:** Baseline characteristics of the patients.

Characteristics	Value
Age, (Mean ± SD, y)	56.5 ± 11.1
**Gender, *N* (%)**	
Male	4 (17.4%)
Female	19 (82.6%)
Baseline mRS, median (interquartile ranges)	0 (0, 0)
**Side of lesion, *N* (%)**	
Right	6 (26.0%)
Left	17 (74.0%)
**Lesion location, *N* (%)**	
Internal carotid artery	20 (87.0%)
Posterior communicating	4
Ophthalmic	12
Carotid-cavernous	4
Vertebral artery (V4 segment)	3 (13.0%)
**Aneurysm morphology, *N* (%)**	
Saccular	20 (87.0%)
Fusiform	3 (13.0%)
**Aneurysm characteristics, (Mean ± SD, mm)**	
Length diameter	8.4 ± 6.3
Neck width	5.3 ± 2.1
**Aneurysm size**	
Small (<10 mm)	15 (65.2%)
Large (10–25 mm)	7 (30.4%)
Giant (>25 mm)	1 (4.3%)

The data are presented in *N* (%) or mean ± standard deviation. SD, Standard deviation; mm, millimeter.

**Figure 2:**
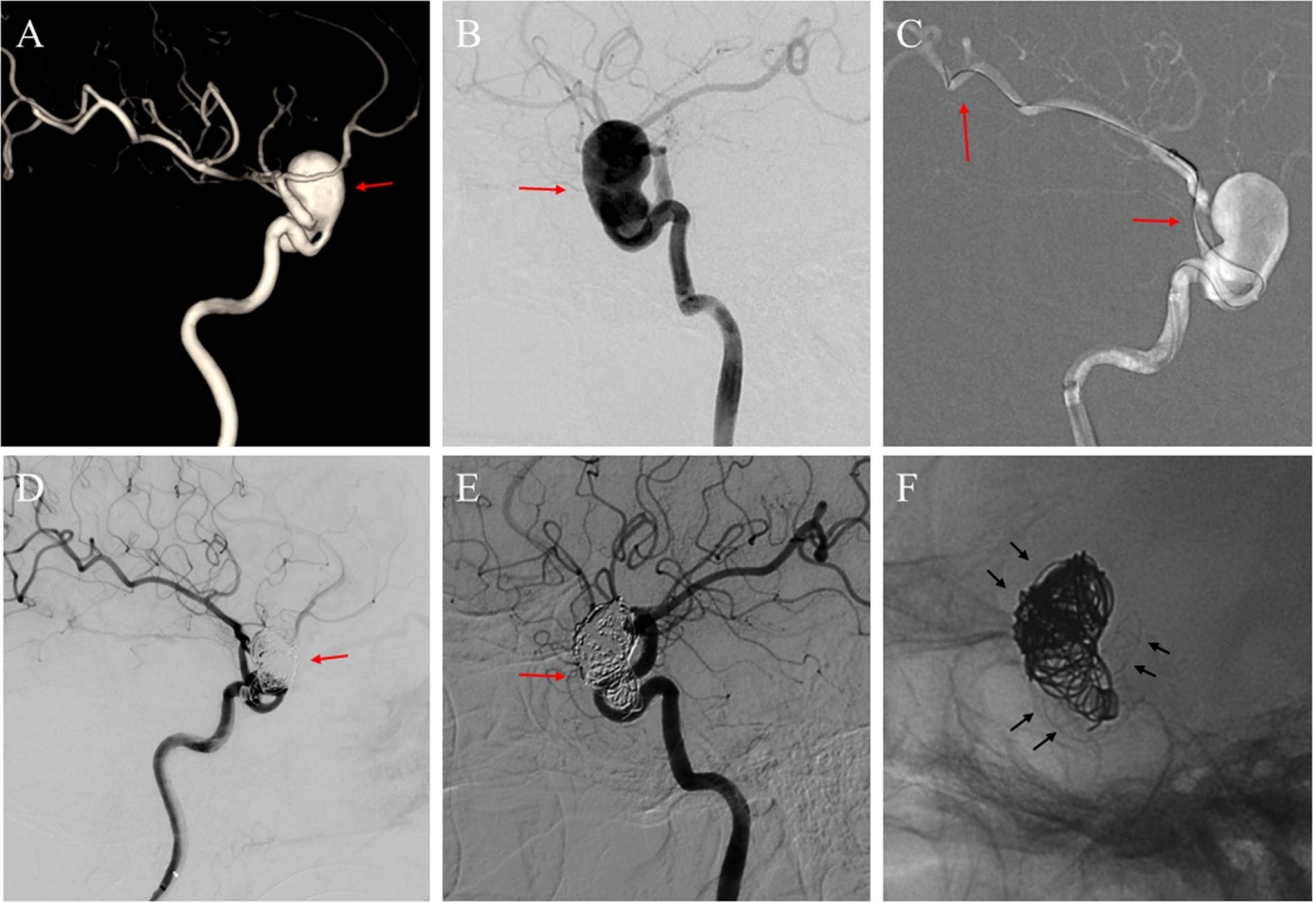
**(A)** A 71-year-old woman with a giant unruptured aneurysm (red arrow) situated in the ophthalmic segment of the left ICA in the three-dimensional reconstruction image; **(B)** The angiography image before the FD implantation procedure illustrated the aneurysm (red arrow); **(C)** A micro-guide wire was employed to navigate a microcatheter, and a distal access catheter was coaxially guided through the aneurysm sac to establish a stable pathway for the delivery and deployment of the FD (red arrows); **(D)** After Choydar FD device (4.0 mm * 30 mm) implantation and the embolization of the coils, a deceleration of contrast agent filling within the aneurysm sac was observed (red arrow); **(E)** DSA image follow-up at 17 months showed the aneurysm located at the ophthalmic segment was completely occluded (red arrow); **(F)** Observation of the Choydar FD device and coils through radiographic imaging (black arrows).

**Figure 3:**
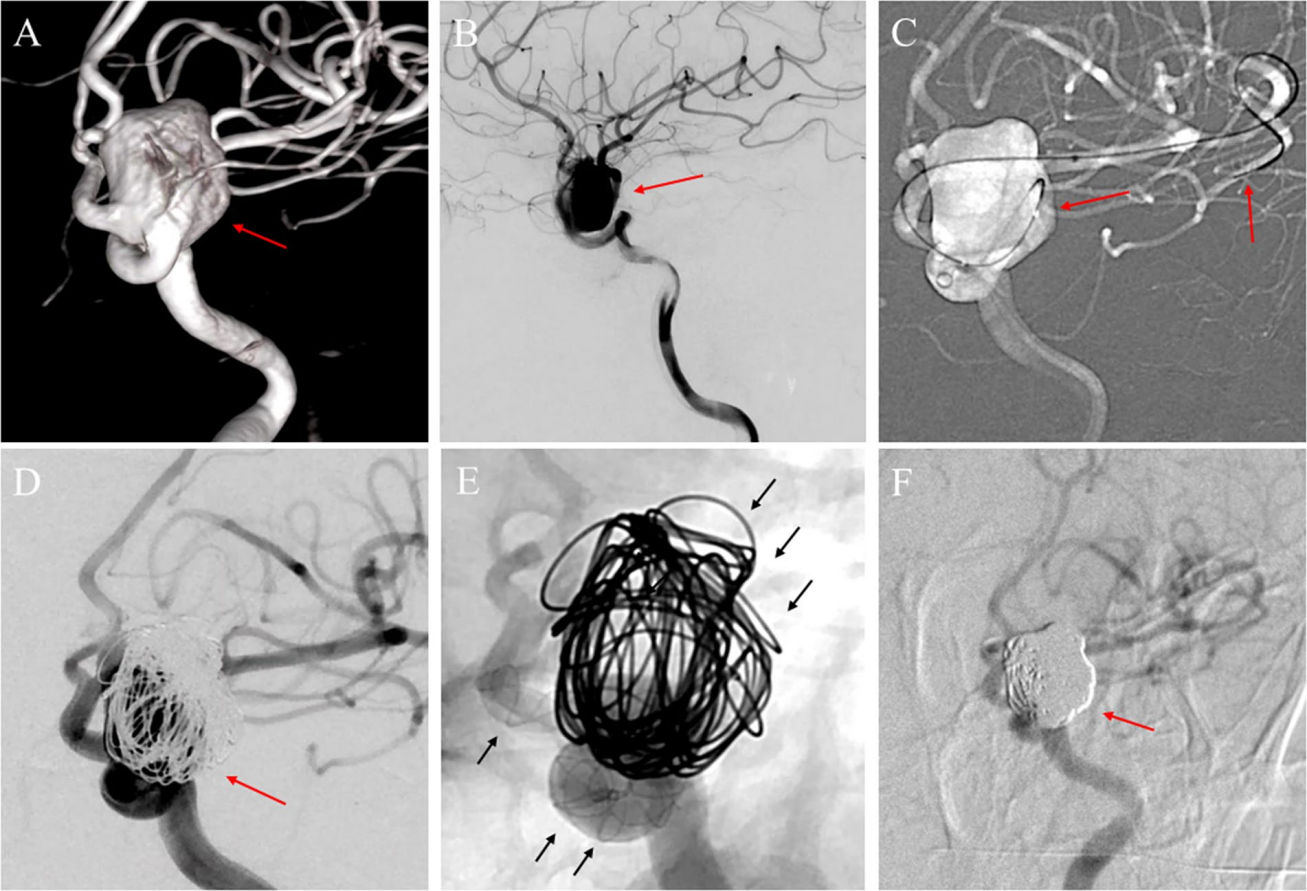
**(A)** A 55-year-old woman with a large unruptured aneurysm (red arrow) identified in the ophthalmic segment of the left internal carotid artery through a three-dimensional reconstruction image; **(B)** The angiography image before treatment presented the aneurysm (red arrow); **(C)** During the procedure, a micro-guide wire was utilized to guide a microcatheter through the aneurysm sac and the parent artery, ultimately reaching the distal end of the middle cerebral artery for deployment of the FD (red arrows); **(D)** Following the implantation of the Choydar FD device (4.75 mm * 30 mm) and the embolization of the coils, a substantial reduction in contrast agent filling within the aneurysm sac was observed (red arrow); **(E)** Illustration of the coils and Choydar FD device in the mask image (black arrows); **(F)** The follow-up imaging with DSA conducted 13 months after the procedure showed the aneurysm previously detected in the ophthalmic segment was completely occluded (red arrow).

Therapeutically, 18 aneurysms were treated with FD devices in combination with coiling, whereas 5 aneurysms received solely FD device deployment. Immediate angiographic results demonstrated that 2 aneurysms received an A rating, 16 a B rating, 2 a C rating, and 3 a D rating, with illustrative cases presented in Figures 2, 3. No new neurological adverse events were reported from the end of the procedure to discharge. Clinical follow-up for all patients indicated favorable outcomes with no neurological adverse events. Angiographic follow-up at an average of 12.1 months post-procedure showed that 22 aneurysms achieved a D rating, and one maintained a C rating. Furthermore, in-stent stenosis was observed in one patient, who did not exhibit ischemic symptoms or neurological deficits, attributed to the efficient collateral circulation within the Circle of Willis. This patient’s care continued with standardized medical therapy. The outcomes are detailed in Table 4.

**Table 4:** Treatment, outcomes, and complications.

Variable	Value
**Microcatheter type for stent delivery, *N* (%)**	
0.021-in ID	7 (30.4%)
0.027-in ID	16 (69.6%)
**Treatment strategy, *N* (%)**	
FD device + coil	18 (78.3%)
FD device	5 (21.7%)
Choydar flow diverter used, *N*	23
Number of devices per patient	1
Successful device deployment, *N* (%)	23 (100%)
**Immediate angiographic outcomes (OKM scale), *N* (%)**	
A	2 (8.7%)
B	16 (69.6%)
C	2 (8.7%)
D	3 (13.0%)
**Clinical outcomes at discharge, *N* (%)**	
mRS 0	23 (100%)
**Perioperative complications, *N* (%)**	
Thromboembolic events	0
Hemorrhage events	0
Follow-up (mean, 12.1 months)	
**Angiographic results (OKM scale), *N* (%)**	
C	1 (4.3%)
D	22 (95.7%)
**Device status, *N* (%)**	
Patency	22 (95.7%)
Stenosis	1 (4.3%)
**Clinical outcomes at follow-up, *N* (%)**	
mRS 0	23 (100%)

The data are presented in *N* (%) or mean. ID, Internal diameter.

## Discussion

In this study, we introduced a newly designed flow divert stent and presented its preliminary application in a cohort of 23 patients. The successful deployment of all devices was achieved, and a commendable 95.7% complete occlusion rate of the aneurysms was accomplished. Furthermore, no adverse events were observed during the follow-up period. The outcomes suggest that the Choydar FD device is a safe and feasible option for the management of intracranial aneurysms.

Since the conception of flow diversion for treating intracranial aneurysms was first proposed by Geremia et al. (9), numerous close-meshed stent systems have been developed and utilized in clinical practice, including the Pipeline embolization device (PED; Medtronic Inc., Minneapolis, Minnesota, United States), the Flow Redirection Endoluminal Device (FRED; MicroVention, Tustin, CA, United States), the p64/p48 Flow Modulation Device (Phenox GmbH, Bochum, Germany), the Tubridge (MicroPort Medical, Shanghai, China), the Silk/Silk Vista Baby (Balt, Montmorency, France), and the Surpass (Stryker Neurovascular, Kalamazoo, Michigan, United States) (10). Initially, FD devices were primarily developed for the treatment of sizeable or colossal aneurysms situated in the petrous to superior hypophyseal segments of the intracranial carotid artery, with subsequent applications encompassing diminutive and intermediate aneurysms extending up to the ICA termination (11). Moreover, certain aneurysms occurring in the posterior circulation, such as those in the vertebral artery, basilar artery trunk, and the bifurcation of the Willis circle, have also been subjected to experimental intervention using FD devices (12–15).

However, one of the major impediments associated with the technology employed for the implantation of FD devices is that most systems need to be delivered with microcatheters featuring an internal diameter of 0.027 in or greater (16). This factor may potentially augment the intricacy of the surgical intervention. An instance of such complexity is the need to accommodate both the stent microcatheter and an additional coiling microcatheter within a single access system of 6 French diameter, which necessitates the operator to perform bilateral femoral artery punctures and establish two access systems. Although previous studies have reported the clinical application of FD devices such as FRED Jr., Silk+, and p48 that are compatible with 0.021-in diameter microcatheters for the treatment of intracranial aneurysms (17), these products have yet to receive certification or enter the market, and their efficacy lacks sufficient supporting evidence within our nation.

To address this issue, the Choydar FD device was developed with corresponding model specifications for varying vessel diameters, offering surgeons additional options when implementing FD combined with a coiling strategy or addressing lesions located in parent arteries with smaller diameters. This design simplifies the surgical procedure by completing the stent implantation and coil filling process. The Choydar FD is compatible with smaller microcatheters (down to 0.017 in), enabling its use in smaller vessels and more complex anatomical configurations, which extends the applicability of the device.

Various studies have documented the feasible results of FD device implantation. The Pipeline for Uncoilable or Failed Aneurysms (PUFS) trial published in 2013 reported a 73.6% occlusion rate at 6-month follow-up (18), which increased to 86.8% at a one-year follow-up (19, 20). Bender MT et al. observed a complete occlusion rate of 82% among 445 aneurysms treated with FD at a mean follow-up of 14 months (21). Luo et al. conducted a multicenter study on a Chinese population and achieved a total of 81.4% complete occlusion among 1,171 patients with 1,322 intracranial aneurysms treated with FD (22). Gariel et al. (23) performed a subgroup analysis of the DIVERSION national prospective cohort study (24), which demonstrated a satisfactory occlusion rate of 82.2% after 1 year of follow-up among cases treated with the Silk flow diverter. Alain Bonafe et al. (25) reported their research on p64, which achieved a complete occlusion rate of 83.7% at a 6-month short-term follow-up among 420 patients. Our study, based on imaging follow-up with an average of 12.1 months, demonstrated a complete occlusion rate of 95.7% for the aneurysms, and further supports the effectiveness of the Choydar FD device for aneurysm treatment.

The occlusion effect of an aneurysm is a primary concern for neurointerventional surgeons during the implantation of a flow-diverting device, as it directly reflects the efficacy of such devices. The current understanding of the mechanisms underlying the use of flow-diverting devices for the treatment of intracranial aneurysms involves the remodeling of the parent arterial lumen and blood flow. Specifically, this involves the application of an appropriate metal coverage braid stent to cover the aneurysm neck, which disrupts direct contact between the parent artery and aneurysm sac, promotes intra-aneurysmal blood stasis and thrombus formation, while also strengthening and restoring the parent artery. Additionally, the gradual process of neointima formation and endothelialization on the surface of the flow-diverting device leads to its complete integration into the wall of the parent artery (26, 27). The prevailing theoretical perspectives posit that improper selection in stent dimensions would have a considerable influence on the porosity of devices. Specifically, the extent of metal coverage is subject to variation in accordance with a relationship between the diameters of the parent artery and the dimension of the FD device. In instances where an FD device is excessively dimensioned, the ensuing effect is a diminution in metal coverage and a concomitant escalation in porosity, which in turn, has the potential to contribute to a decrease in the rate of aneurysm occlusion (17).

In response to this concern, the Choydar device has been developed with diverse specifications incorporating cerebral vessel diameters ranging from 2 to 6.5 mm. This enables surgeons to select appropriate devices during the operation according to the precise diameter of the parent artery at the lesion site, thereby conferring potential benefits for favorable radiological outcomes.

The Choydar FD device offers several advancements over previous flow diverters, which enhance its performance and safety profile.

### Distal closed-cell structure

Unlike other flow diverters with open-cell designs, the Choydar FD features a closed-cell structure at its distal end. This design enhances the stability and apposition to the vessel wall, reducing the risk of migration and improving aneurysm occlusion. The closed-cell structure helps to minimize the chances of stent migration and ensures a more secure placement within the vessel.

### Material composition

The Choydar FD is composed of nitinol and platinum-tungsten alloy. The nitinol provides excellent conformability and radial strength, allowing the stent to adapt to the vessel’s contours. The platinum-tungsten alloy enhances the stent’s visibility under fluoroscopy, facilitating accurate placement and assessment of stent expansion during the procedure. This combination of materials ensures both flexibility and durability, crucial for long-term efficacy.

### Enhanced radiopacity

The stent incorporates four platinum-tungsten alloy strands intertwined with nitinol, providing full-body radiopacity. This “edge-to-edge” radiopaque design allows for clear visualization of the stent’s position, degree of opening, and apposition to the vessel wall. Enhanced radiopacity is critical for ensuring precise deployment and immediate post-deployment assessment, reducing the likelihood of complications due to improper placement.

### Adaptability to smaller vessels

The Choydar FD is compatible with smaller microcatheters (down to 0.017 in), enabling its use in smaller vessels and more complex anatomical configurations. This adaptability simplifies the procedure and extends the applicability of the device to a broader range of aneurysm cases. The ability to use smaller microcatheters also reduces the procedural invasiveness and potentially decreases the risk of vessel damage.

### High metal coverage

The Choydar FD offers a high metal coverage rate (30–35%), which is crucial for effective flow diversion and aneurysm occlusion. This high coverage is achieved through a dense braided design that ensures comprehensive coverage of the aneurysm neck. High metal coverage improves the likelihood of successful aneurysm occlusion by promoting blood flow redirection and thrombus formation within the aneurysm.

The PUFS trial reported a perioperative ischemic stroke rate of 2.8% (3/107) and a rate of 1.9% (2/107) in ipsilateral hemorrhage, with 3 patients dying within the study cohort. Luo et al. presented an ischemic stroke rate of 7.3% (85/1,171), an SAH rate of 2.0% (24/1171), ipsilateral post-procedure hemorrhage rate of 2.0% (23/1,171), and mortality rate of 1.5% (17/1,171) (22). Lylyk et al. (28) documented a rate of 3.6% (30/835) in ischemic stroke, 1.4% (12/835) in aneurysm rupture, and 0.4% (3/835) in intraparenchymal hemorrhage, with a rate of 4.6% in all-cause mortality. Bhatia et al. (29) conducted a systematic review and meta-analysis, focused on periprocedural complications of the second-generation FD treatment, and illustrated a major ischemic stroke rate of 0.4% (4/901) and a major hemorrhage rate of 1.0% (9/901), with a total mortality rate of 0.8%. Another meta-analysis by Lv et al. (30) analyzed the rates of procedure-related complications, including both ischemic and hemorrhagic events, which are critical factors influencing the clinical outcomes of patients treated with FD devices. These findings underscore the necessity for ongoing efforts to improve patient outcomes in those who have undergone FD implantation.

Currently, some studies have been conducted on the underlying mechanisms of complications that arise from the implantation of FD devices, such as the foreign materials commonly employed in the coating of interventional devices, resulting in initial ischemic lesions and hemorrhagic transformation (31). Additionally, there are fluctuations in the blood flow of the parent artery, distal collaterals, and intra-aneurysm blood flow that may occur following FD implantation. Furthermore, thrombus formation in the aneurysm sac and thrombus-induced autolysis, which includes the infiltration of the aneurysm wall with inflammatory cells and enzymatic degradation, may increase the risk of hemorrhage complications (27, 32, 33). On the other hand, factors such as antiplatelet aggregation strategies, duration of surgery, and procedural complexity may contribute to ischemic complications (34–36). In the course of our investigation, the absence of hemorrhagic or ischemic incidents reinforced the safety profile of the Choydar FD device. The minimal occurrence of adverse events within our cohort may be ascribed to meticulous patient selection and the judicious determination of FD specifications. In addition, the relatively high proportion of patients who underwent a strategy of FD implantation combined with coil embolization was also one of the factors contributing to the reduction of bleeding complications (26). Furthermore, the features of Choydar FD device, including nitinol braided wire as the main material that enhances resistance to bending and twisting (37), and the addition of platinum-tungsten alloy to improve radiopacity, provide benefits for surgeons to observe whether the stent has expanded and is well apposed to the vessel wall during the operation. Meanwhile, the distal edge of the stent is designed as a closed structure, which helps reduce the risk of damaging the vessel wall during the pushing and pulling techniques within stent deployment.

To the best of our understanding, this investigation constitutes the first report concerning a novel FD device in our country. However, it is imperative to note that this study serves as an exploratory analysis with a relatively limited sample size. Furthermore, the study’s design is uncontrolled, lacking a comparative arm, which may affect the robustness of the conclusions drawn. Additionally, despite the device’s design accommodating a 0.017-in microcatheter system for stent delivery, the specific model intended for this application was not utilized within our study due to the lack of suitable cases. This omission constitutes a regrettable limitation of our investigation.

In conclusion, we presented the structure and configuration of the Choydar FD device and illustrated our preliminary experience. Our findings suggest that the Choydar FD device is a promising option for treating intracranial aneurysms, as it demonstrates satisfactory efficacy and safety. Further investigations comprising extensive large-scale and long-term follow-up assessments are warranted to authenticate these preliminary outcomes.

## Data availability statement

The original contributions presented in the study are included in the article/supplementary material, further inquiries can be directed to the corresponding author.

## Ethics statement

The studies involving humans were approved by Institutional Review Board of Union Hospital, Tongji Medical College, Huazhong University of Science and Technology. The studies were conducted in accordance with the local legislation and institutional requirements. The participants provided their written informed consent to participate in this study.

## Author contributions

CL: Writing – original draft. YC: Writing – review & editing. CZ: Formal analysis, Writing – review & editing. KG: Methodology, Writing – review & editing. XH: Writing – review & editing.
